# Diversity of Potentially Pathogenic *Escherichia coli* O104 and O9 Serogroups Isolated before 2011 from Fecal Samples from Children from Different Geographic Regions

**DOI:** 10.3390/microorganisms9112227

**Published:** 2021-10-26

**Authors:** Armando Navarro, Claudia van der Ploeg, Ariel Rogé, Delia Licona-Moreno, Gabriela Delgado, Rosario Morales-Espinosa, Alejandro Cravioto, Carlos Eslava

**Affiliations:** 1 Public Health Department, Faculty of Medicine, Universidad Nacional Autónoma de México, Avenida Universidad 3000, Ciudad Universitaria, México City 04510, Mexico; liconam@unam.mx; 2 Servicio de Antígenos y Antisueros, Instituto Nacional de Producción de Biológicos (INPB)—ANLIS “Dr. Carlos G. Malbrán”, Buenos Aires 1282, Argentina; claudiaavdp@gmail.com (C.v.d.P.); aroge@anlis.gob.ar (A.R.); 3 Departamento de Microbiología y Parasitología, Facultad de Medicina, Universidad Nacional Autónoma de México, México City 04510, Mexico; delgados@unam.mx (G.D.); marosari@unam.mx (R.M.-E.); 4 Faculty of Medicine, Universidad Nacional Autónoma de México, México City 04510, Mexico; dracravioto@hotmail.com; 5 Peripheral Unit of Basic and Clinical Research in Infectious Diseases, Public Health Department, Research Division, Faculty of Medicine Universidad Nacional Autónoma de México, Bacterial Pathogenicity Laboratory, Hemato-Oncology and Research Unit, Children’s Hospital of Mexico Federico Gómez, Dr. Márquez 162, Col. De los Doctores, México City 06720, Mexico; carlos_01eslava@yahoo.com.mx

**Keywords:** *Escherichia coli* O104:H4, serotypes, virulence genes, STEAEC, DEC pathotypes

## Abstract

In 2011, an outbreak of hemorrhagic colitis and hemolytic uremic syndrome (HUS) was reported in Europe that was related to a hybrid STEAEC of *Escherichia coli* (*E. coli*) O104:H4 strain. The current study aimed to analyze strains of *E. coli* O104 and O9 isolated before 2011. The study included 47 strains isolated from children with and without diarrhea between 1986 and 2009 from different geographic regions, as well as seven reference strains. Serotyping was carried out on 188 anti-O and 53 anti-H sera. PCR was used to identify DEC genes and phylogenetic groups. Resistance profiles to antimicrobials were determined by diffusion in agar, while PFGE was used to analyze genomic similarity. Five serotypes of *E. coli* O104 and nine of O9 were identified, as well as an antigenic cross-reaction with one anti-*E. coli* O9 serum. *E. coli* O104 and O9 presented diarrheagenic *E*. *coli* (DEC) genes in different combinations and were located in commensal phylogenetic groups with different antimicrobial resistance. PFGE showed that O104:H4 and O9:(H4, NM) strains from SSI, Bangladesh and México belong to a diverse group located in the same subgroup. *E. coli* O104 and O9 were classified as commensal strains containing DEC genes. The groups were genetically diverse with pathogenic potential making continued epidemiologic surveillance important.

## 1. Introduction

In 2011, an outbreak of hemorrhagic colitis (HC) and hemolytic uremic syndrome (HUS) was reported in various European countries [1]. Those affected were adults over the age of 20 years and importantly, women were affected in greater numbers than men [2]. According to a preliminary report, more than four thousand cases and fifty deaths were registered [3]. While searching for the agent responsible for the outbreak, a strain of *Escherichia coli* (*E*. *coli*) STEC no-O157 of the O104:H4 serotype was identified. Genetic analysis of this strain showed the presence of the *aatA*, *aggR*, *aap*, *agg*, and *aggC* genes of enteroaggregative *E*. *coli* (EAEC) and *stx2* of Shiga toxin-producing *E*. *coli* (STEC), leading to the notion that this was an STEAEC hybrid strain [4,5,6]. The term EAHEC was also proposed for strains that contain these genes, and they were identified as being LEE-negative [7]. The emergence of this hybrid bacteria suggests an uncommon genetic recombination event, although some time before the reported outbreak in Germany in 2011, the participation of EAEC in HUS cases, including some O104 serogroup strains, had been reported [8,9]. The aforementioned outbreak reached other countries, such as Austria, Denmark, Germany, Holland, Norway, Spain, Sweden, Switzerland and England. In addition, cases were reported in individuals who had previously traveled to the region in Germany in which the outbreak originated [10]. The global report by the European Food Safety Authority reported that 16 European countries had been affected, as well as the United States of America [11]. Cases were also reported in individuals in Canada and the United States who had visited Germany some days before becoming sick [10]. The source of the outbreak caused by *E*. *coli* O104:H4 initially implicated cabbages, tomatoes, vegetable salad and cucumbers consumed raw. In fact, epidemiological evidence suggested that contaminated fenugreek seeds were the source of the outbreak [12,13,14].

One of the methods for characterizing *E*. *coli* strains is serological typing proposed by Kaufmann in the 1940s [15]. For a long time, this method was considered to be the gold standard to determine the antigenic characteristics of bacteria. Serological typing has been the standard tool for taxonomic and epidemiologic studies to characterize *E. coli* isolates during epidemic outbreaks associated with this bacterium. Serologic typing also allows new serogroup clusters to be established using DNA sequencing methodologies gene groups synthesized by the O antigen (O-antigen gene cluster (O-AGC)), or complete genome sequencing to develop genotypic methods to determine O antigen groups [16,17,18].

In our own laboratory, we carried out systematic *E*. *coli* typing using sera from rabbits prepared against 188 somatic antigens (O) and 56 flagella (H) according to the method reported by Ørskov [19]. While carrying out these studies, we identified antigenic reactivity shared between the O104 and O9 serogroup strains, suggesting that both serogroups could belong to the same clone. Given the epidemiologic importance that the O104 serogroup acquired, we carried out a review of our database in order to determine the isolation frequency of the *E. coli* O104 and O9 obtained from different epidemiologic studies carried out in our laboratory [20]. The results from this review showed that we housed isolates from Mexico, Egypt, Bangladesh and Argentina, although in Mexico there were no reports referring to HUS or HC related to *E. coli* O104. However, the existence of this microorganism in countries in which HUS and HC is not a public health problem suggests that the genotypic and phenotypic characteristics of the bacteria are unknown. It is for this reason that the current study looked at the antigenic cross-reaction between *E. coli* O104 and O9, the presence of diarrheagenic *E*. *coli* (DEC) gene groups that are associated with the pathogenesis of diarrhea in HUS and HC, and the potential clonal association of O104 and O9 strains in order to identify its epidemiologic impact, as observed in the 2011 outbreak in some European Union countries.

## 2. Materials and Methods

### 2.1. E. coli Strains

Of the 54 *E*. *coli* strains analyzed, 47 were fecal samples from 35 children under five years of age (30 with diarrhea and 5 without symptoms) obtained from epidemiologic studies carried out in Mexico [20], Egypt, Argentina and Bangladesh (obtained from International Centre for Diarrhoeal Disease Research, Bangladesh, ICDDR,B). In addition to these strains, four *E*. *coli* reference strains were included in the analysis (ECOR16, ECOR26, ECOR27, ECOR28), which were provided by Dr. Robert Selander in 1985 [21] (20), two *E*. *coli* O104:H4 strains obtained from the Statens Serum Institut (SSI) in 2013 and 2018 [22,23], and one O9:H12 (Bi3 16-42) strain from the Gastrointestinal Bacteria Reference Unit (GBRU), Public Health England, London, UK.

### 2.2. Serological Typing

In order to confirm the serotypes of the selected *E*. *coli* strains, serological typing used 188 sera (SERUNAM, Mexico) prepared against the somatic (O) antigen and 53 against the flagellar (H) antigen according to the method reported previously by Ørskov [19].

### 2.3. Antigenic Reactivity Compared between E. coli O104 and O9

Absorption of anti-O104 and O9 antibodies with corresponding antigens (O104 and O9) was carried out in the laboratory using the method described by Ewing [24] with minor modifications [25]. Using the absorbed sera, the antigenic relationship of *E*. *coli* O104 (H519) and O9 (Bi316-42) strains was analyzed by microagglutination test [19]. For this, the dilution at which each sera agglutinated was determined.

### 2.4. Detection of Virulence Genes

Using PCR and specific primers (Table 1), the presence of *eae*, *stx1*, *stx2*, *hlyA*, *aggR*, *aapA*, *aatA*, *aaiC*, *sat* and *fimH* genes present in the STEC, EAEC and UPEC pathotypes of *E. coli* were identified.

### 2.5. Phylogenetic Group

Phylogenetic groups (A, B1, B2, C, D, E, F and *Escherichia* cryptic clade I) of *E. coli* O104 y O9 were determined using PCR with primers for *arpA*, *chuA*, *yjaA* and *TspE4.C2* (Table 1) and previously reported conditions [34].

### 2.6. Antimicrobial Sensitivity

Sensitivity tests used the procedures and recommendations proposed in the Clinical & Laboratory Standards Institute (CLSI) 2017 manual [35]. Bacteria were grown on a nutrient agar plate and incubated at 37 °C for 24 h. A suspension of the resulting bacterial growth with a saline solution was prepared and adjusted to a 0.5 nephelometric McFarland tube (1.5 × 10^8^ bacteria/mL). The suspension was inoculated into 2 Mueller-Hinton agar plates. Disks (BBL Sensi Disc BD^®^, Franklin Lakes, NJ, USA) with the following antimicrobials were placed on the surface of the inoculated agar plates at a distance of 24 mm between each: Cephalosporins 2nd generation: cefoxitin (FOX) 30 μg; 3rd generation: ceftriaxone (CRO) 30 μg, ceftazidime (CAZ) 30 μg, cefotaxime (CTX) 30 μg; 4th generation: cefepime (FEP) 30 μg; quinolones: ofloxacin (OFX) 5 μg, norfloxacin (NOR) 10 μg, nalidixic acid (NA) 30 μg, ciprofloxacin (CIP) 5 μg, imipenem (IPM) 10 μg, aztreonam (ATM) 30 μg, trimethoprim/sulfamethoxazole (SXT) 1.25/23.75 μg, tetracycline (TE) 30 μg. The plates were incubated at 37 °C for 16–18 h, and then the inhibition halos were measured to determine strain behavior. The tests used 25922 and 35218 reference strains from the American Type Culture Collection (ATCC).

### 2.7. Chromosomal Profiles by Pulse Field Gel Electrophoresis (PFGE)

Genomic DNA in agarose blocks was prepared using the method previously described in PulseNet (https://www.cdc.gov/pulsenet/participants/international/index.html (accessed on 18 September 2021)) with some modifications that included allowing bacterial growth for no more than 12 h, deproteinizing the DNA-plugs of the bacterial colony twice and increasing the number of washes (up to 8) of the DNA-plugs with TBE buffer. The XbaI (Sigma-Aldrich, St. Louis, MO, USA) enzyme was used to obtain chromosomal profiles. XbaI fragments were separated by a CHEF-Mapper device (Bio-Rad, Hercules, CA, USA). *Salmonella* Braenderup H9812 DNA restricted by XbaI was used as a molecular size marker. The gels were run at 12 °C, 6 V/cm and with a 120° switch angle for 19 h with a pulse time that ramped up from 2.16 s to 54.17 s. Following resolution and staining with ethidium bromide, each profile was viewed on a 1.0% agarose gel (Seakem Gold agarose, Lonza Rockland, Rockland, ME, USA).

The images were digitized by the Gel Logic 112 imaging system (Kodak, Rochester, NY, USA). The fingerprinting profile in the PFGE gel was analyzed using BioNumerics v.7.1 (AppliedMaths, Sint-Martens-Latem, Belgium) software package. After background subtraction and gel normalization, typing of fingerprint profiles was carried out, which was based on banding similarity and dissimilarity, using the Dice similarity coefficient and the Unweighted Pair Group Method with Arithmetic Mean (UPGMA) [36] according to average linkage clustering methods.

## 3. Results

### 3.1. Origin of the Strains

Of the 54 analyzed *E. coli* strains, 47 were obtained from clinical isolates, of which 5 (10.6%) were from Egypt, 4 (8.5%) from Argentina, 3 (6.4%) from Bangladesh and 35 (74.5 %) from Mexico. 

### 3.2. Antigenic Elements Shared between E. coli O104 and O9

In order to ascertain that the agglutination assays with anti-O9 and anti-O104 sera were identifying the specific serogroup, absorption tests of both antisera using the heterologous antigen were carried out. The reactivity of anti-*E. coli* O9 serum against O9 and O104 antigens showed responses at dilutions of 1:1600 and 1:400, respectively. The same assay for the anti-*E. coli* O104 serum against O9 and O104 antigens showed a response at a dilution of 1:200 and 1:1600, respectively (Supplementary Table S1). The reactivity of the anti-O9 serum absorbed with the O104 antigen showed that reaction against this antigen was removed. Regarding the reactivity of the anti-O104 serum absorbed with the O9 antigen, reaction against the O104 antigen was eliminated. Following these results, absorbed antisera were used for each serogroup. The strains from the O104 serogroup showed agglutination response with its specific serum (anti-*E. coli* O104 serum), a dilution of 1:400 without presenting any response against the specific anti-*E. coli* O9 serum. Interestingly, the O9 serogroup strains reacted only to the specific anti-*E*. *coli* O9 serum.

### 3.3. Serotypes, Phylogenetic Groups, Virulence Gene Content and Pathotypes of E. coli O104 Strains

Serologic tests of the 47 clinically isolated strains showed that 13 corresponded to the O104 serogroup with the following serotypes: O104:H4 (38%), O104:H7 (8%) and O104:H21 (54%). The phylogenetic tests showed the strains belonged to phylogroups of commensal strains A (69%) and B1 (31%). PCR was used to identify virulence-associated genes (Table 2) detected a-EPEC/EAEC (7.7%), STEC (61.5%) and combinations of STEC/EAEC (30.8%) in the strains.

### 3.4. Serotypes, Phylogenetic Groups and Pathotypes of E. coli O9 Strains

From a total of 34 strains belonging to serogroup O9, the following distribution of serotypes was found: O9:NM (nonmotile) (35.3%), O9:H4 (11.8%), O9:H9 (8.8%), O9:H10 (2.9%), O9:H11 (5.9%), O9:H21 (2.9%), O9:H25 (20.6%) and O9:H33 (11.8%). The majority (91%) of these belonged to the phylogroups defined as commensal A (62%) and B1 (29%), and with less frequency to the extraintestinal pathogenic B2 (3%) and C (6%). The main virulence genes detected were EAEC (34%), STEC (35%), STEC/EAEC (32%) and a-EPEC (6%), with no amplification being found in 3% of strains (Table 3).

### 3.5. Serotypes, Phylogenetic Groups and Genes Associated with Virulence in Reference Strains

Of the serotypes identified in the reference strains, two were O104:H4, one O104:H2 and two O104:H21, which belong to the phylogenetic groups A, D and B1. In these strains, the following genes were present: one *stx1*/*hlyA* (STEC), three with EAEC genes, one *eae*/*hlyA* (a-EPEC) and two not determined (ND) (Table 4). The O9 serogroup reference strains corresponded to O9:H12 from the GBRU collection within phylogenetic group A with *aggR* of EAEC. The other strain from the ECOR16 collection was serotype O9:H10 from phylogroup A that amplified *fimH* (Table 4).

### 3.6. Sensitivity to Antimicrobials

Results from the sensitivity tests to various antimicrobials for the O104 and SSI strains showed that three (30%) were resistant to only one microbial (TE, SXT or NA), four (40%) to NA/TE and one (10%) to ATM/CAZ/NA (Table 5).

Results for the O9 strains showed resistance in 21 (62%) of the strains and of these, 13 were resistant to just one antimicrobial (SXT 3 (9%), TE 9 (27%) and ATM 1 (3%)) and eight were resistant to two antimicrobials (NA/TE 2 (6%) and TE/SXT 6 (18%)) (Table 6). The ECOR16, ECOR26, ECOR28 and O9:H12 (Bi3 16-42) strains were sensitive to all antimicrobials. 

### 3.7. Pulsed-Field Gel Electrophoresis (PFGE)

**Figure 1:**
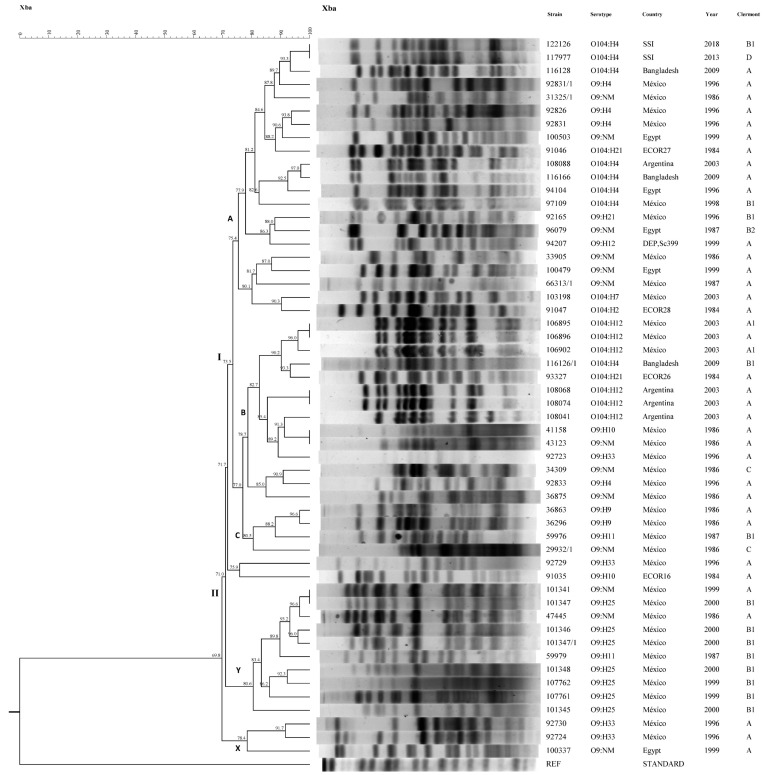
Pulsed-field gel electrophoresis (PFGE). NM: nonmotile.

Using PFGE (Figure 1), 49 electrophoretic types were seen that formed two main branches (I and II). In branch I, the largest number of strains grouped together to form three clusters (A, B and C). Of these, cluster A was made up of five subgroups. In the first subgroup, two O104:H4 strains isolated in 2013 and 2018 from the SSI were located, and both strains were positive for *aggR* (EAEC) and *sat*, which are both genes present on the DEC strains of the EAEC pathogens. In this subgroup, an O104:H4 strain isolated in Bangladesh (2009) also presented *eae*/*aggR*/*stx1*/*sat* genes, and the similarity between this strain and the two from the SSI was 93.3%. Two strains isolated in Mexico were also present in this first subgroup. One of the Mexican strains was O9:H4 and presented *aggR*/*stx1*/*fimH*, while the other, which was an O9:NM strain isolated in 1986, presented *stx1* and and was *fimH*-positive. The similarity between these five strains was 89.7%. 

Two O9:H4 strains isolated in Mexico and positive for *aggR*/*stx1*/*fimH*, and one O9:NM strain isolated in Egypt in 1999 harboring *aggR*/*fimH*, belonged to the second subgroup. An O104:H21 (ECOR27) strain isolated in 1984 was also located in this subgroup and presented *stx1*/*fimH*. The similarity between these strains was 88.2%.

The third subgroup comprised four O104:H4 strains: one strain from Argentina isolated in 2003, two strains from Bangladesh isolated in 1996 and 2009, and one strain from Mexico isolated in 1998. The similarity between these four strains was 82.6%. The strain from Argentina harbored *eae*/*stx1*/sat/*fimH*, the strains from Egypt and Bangladesh were positive for *eae*/*aggR*/*stx1*sat and the isolate from Mexico presented *eae*/*aggR*/*fimH*.

In the fourth subgroup, serotypes O9:H21 from Mexico isolated in 1996 and O9:NM from Egypt isolated in 1987 harbored *aggR* and *fimH* genes. A strain of O9:H12 (sc399) that presented *aggR* and *fimH* also belonged to this subgroup. The similarity between members of the fourth subgroup was 86.3%.

Five strains belonged to subgroup five. Three were serotype O9:NM, two of which were isolated in Mexico in 1986 and 1987, while the third was isolated in Egypt in 1999. The fourth strain was an O104:H7 strain isolated in Mexico in 2003 and presented *eae*/*stx1*/*fimH* genes, and the final strain was O104:H2 (ECOR28) that had no virulence genes (ND). The similarity between these five strains was 80.1%. 

Cluster B comprised three subgroups. In the first subgroup, there were three strains of O104:H12 isolated in Mexico in 2003 that were positive for *eae*/*stx1*/*stx2*/*fimH*, one O104:H4 strain from Bangladesh isolated in 2009 that presented *aggR*/*eae*/*stx1*, and one O104:H21 (ECOR26) strain that harbored *eae*/*fimH*. The second subgroup was made up of three O104:H12 strains isolated in Argentina in 2003, all of which were positive for *eae*, *stx1*/*stx2*/*fimH*. The third subgroup was formed by an O104:H12 strain isolated in Argentina in 2003 that presented *eae*/*stx1*/stx2/*fimH*, and three strains isolated in Mexico, O9:H10 and O9:NM both isolated in 1986, and O9:H33 isolated in 1996, which harbored *aggR*/*stx1*/*fimH*, *aggR*/*fimH* and *eae*/*stx1*/sat/*fimH*, respectively. The similarity between these strains was 89.2%.

Eight strains isolated in Mexico in 1986, 1987 and 1996 grouped into cluster C with the following serotypes: O9:NM, O9:H4, O9:H9, O9:H10, O9:H11 and O9:H33. Of these, four presented *stx1*/*fimH*: two *aggR*/*stx1*/*fimH*, one *sat*/*fimH* and one *eae*/*sat*/*fimH.* One strain belonged to an isolated group, namely O9:H10 (ECOR16), with no virulence genes (ND) and a similarity of 71.7% in branch I. 

Branch II contained two clusters identified as X and Y. In the latter Y cluster, there were ten strains from Mexico isolated in 1986, 1999 and 2000 with serotypes O9:NM, O9:H11 and O9:H25. Of these, four were positive for *aggR*/*stx1*/*fimH*, another four for *aggR*/*fimH*, one presented *stx1*/sat/*fimH*, and the final strain presented *stx1*/*stx2*/*fimH*. Similarity between strains of the Y cluster was between 80.6% and 100%. Cluster X showed lower similarity (69.8%) with branches I and II and included two O9:H33 strains from Mexico isolated in 1996, one which was positive for *eae*/sat/*fimH* and the other for *eae*/*stx1*/sat/*fimH*, and an O9:NM from Egypt isolated in 1999, which presented *aggR*/*stx1*/sat/*fimH*. The similarity between the two Mexican strains was 91.7% and between those and the strain from Egypt was 78.4%.

## 4. Discussion

STEC and EAEC are important pathogens; the former causes HUS and the latter persistent diarrhea. In 2011, a new hybrid variant emerged in European countries that was a strain of the *E. coli* O104:H4 serotype that presented genes of both the STEC and EAEC pathotypes [1]. This current study provides data for *E*. *coli* strains of serotype O9 and O104 isolated in different regions and at different times before the HUS outbreak in Europe in 2011 that was caused by *E*. *coli* O104:H4. We isolated *E. coli* strains from children under five years of age with and without diarrhea, whose fecal samples were characterized by presenting the *stx1*-*stx2* genes in combination with EAEC genes.

Antigenic cross-reactions between O9 and O104 antigens. Analysis of the absorption tests for anti-*E*. *coli* O9 and anti-*E*. *coli* O104 showed that antigenic cross-reactions were eliminated. Results also revealed the presence of common epitopes between both antigens. Previous studies have reported that the LPS of *E*. *coli* O104 presents a structural similarity to the K9 capsular antigens of *E*. *coli* O9 [19,37,38]. Bearing this in mind, the antigenic reactions in the *E*. *coli* O9 and O104 strains of this study could be due to common epitopes between these two LPSs. Such antigenic cross-reactions have been reported in human serum and cow’s milk [39,40]. This is relevant because it provides a starting point to look for common epitopes between the different antigens in order to develop protective vaccines against these pathogens.

Serotypes and virulence genes, phylogenetic groups and diarrheagenic groups of *E. coli* O104 strains. Serotyping analysis showed the presence of three serotypes for *E. coli* O104 (H4, H7 and H12), with O104:H4 being the most frequent. These strains were isolated in Mexico, Egypt and Bangladesh before 2011. An interesting characteristic of these strains was that they presented virulence factors similar to the European O104:H4 strains and were identified as STEC, STEC/EAEC and aEPEC/EAEC hybrids. One microbiological property of the *E. coli* O104:H4 strains isolated during the outbreak in 2011 is that they presented *stx2* and *aggR* genes. However, *E*. *coli* O104 strains of the present study harbored STEC genes in different combinations. In the Egyptian and Bangladeshi strains, *eae* was detected in combination with *stx1*, *aggR*, *aap* and *aatA*. This last characteristic corresponds with the *E. coli* O104:H4 strains (*aggR*, *aap* and *aatA*) from Germany [4]. The presence of the *eae* gene in our study strains is not only a difference from the O104:H4 strains from the outbreak in Germany but also from other O104 strains isolated from cow’s milk and various sources for which the absence of the *eae* gene has been reported [41,42]. The two O104:H4 strains obtained from the SSI were found in the EAEC group based on the presence of *aggR*, *aatA* and *aaiC* genes and the absence of *stx1* and *stx2* genes. The diarrheagenic groups identified in the SSI strains correspond to O104 strains from Mexico, Egypt and Bangladesh in that they contained the EAEC genes (*aggR*, *aatA* and *aaiC*). However, there was a difference since our study strains presented the *eae* gene, which led them to be grouped as STEC/EAEC and a-EPEC/EAEC. The lack of the *stx1* and *stx2* genes in the O104:H4 strains from the SSI was an important difference with respect to the 2011 epidemic outbreak in Europe. However, the genes in the SSI strains corresponded to that reported in the fourth and eighth External Quality Assessment (4th EQA and 8th EQA) carried out by the SSI in 2013 and 2018 [22,23].

Another interesting serotype identified in our study was *E*. *coli* O104:H7. This serotype presented the *eae* gene and for this reason, it was classified as atypical EPEC (a-EPEC), which is a difference from other strains with the same serotype isolated from cases of human diarrhea and sheep that were reported as being positive for *stx* and negative for *eae* [41,42,43]. With this in mind, it has been proposed that cattle and sheep could be possible reservoirs for O104:H7 [41,43]. Another serotype identified in our study was O104:H12 from Mexico, Argentina and Bangladesh, which was classified as STEC, and STEC/EAEC. This serotype has been reported as being present in rectal swabs of cattle, but without the *stx* gene [40,41]. In contrast, in our study strains, the *stx1* and *eae* genes were detected indicating the diverse genotypes that can be found in O104 strains.

Serologic typing of the ECOR26, ECOR27 and ECOR28 strains identified two serotypes, namely O104:H21 and O104:H2. These results are in line with those reported by Amor and Johnson [44,45] and initially reported by T Whittam in the Thomas Whittam Laboratory website (http://www.bio.psu.edu/People/Faculty/Whittam/Lab/ecor/ (accessed on 18 September 2021) [45]). This shows that the typing of *E*. *coli* using 188 anti-O sera continues to be valid for providing knowledge of antigenic characteristics of different strain collections and origins.

A similar situation to that of the *E*. *coli* O104 serotypes was observed in *E*. *coli* O9 serotypes in that the serotypes isolated in Mexico according to gene presence were classified as STEC, and STEC/EAEC pathotypes. These characteristics correspond to STEC strains isolated from healthy pigs and O9:NM strains from human infection [46,47,48]. However, these strains classified as STEC differ from O9 strains isolated from dairy cattle from different parts of Mexico in that the *stx1* genes were not identified, although they did share the genes of the EAEC pathotype [40].

Some bacterial structures, such as adhesins and more specifically FimH, have been related to adherence to human epithelial cells, which allows the persistence of bacteria in the intestine. We explored our study strains for the presence of the specific adhesin mannose (*fimH*) of *E*. *coli*. Interestingly, serogroup O104 as well as serogroup O9 presented the *fimH* gene, which confers with the study reported by Shridhar [41] in which strains of the O104:H7 serotype isolated from both humans and cattle presented the *fimH* gene. However, the role of this gene is controversial, given that it has been found as much in virulent strains as in commensal strains [49,50], but there is no doubt that it does play a role in the initial colonization of the human intestine, and as with adhesin, it favors epithelial cell adherence of the intestine and urinary tract.

Phylogenetic groups. The serotypes of the O9 and O104 serogroups of this study belong mainly to phylogroups A and B1, which are classified as commensal bacteria, and they form the normal microbiota of human, pig and bovine intestine [51,52]. Due to these *E*. *coli* strains carrying STEC and EAEC genes, they could be considered as hybrid strains, as was the case of *E*. *coli* O104:H4 isolated from the 2011 epidemic in Germany [53]. However, the O104:H4 strains isolated in Mexico, Bangladesh and a percentage isolated by the SSI were located in phylogenetic group B1, a characteristic that corresponds to strains in the German HUS outbreak, as well as to strains from other studies in which they were located in the same B1 phylogroup [54,55,56]. However, while O104 strains were located in groups considered to be commensal according to the Clermont system [34], other authors report that the presence of virulence factors may not correlate with phylogenetic groups of *E*. *coli* [57]. It is for this reason that the presence of *E*. *coli* strains with virulence factors in commensal phylogroups is a frequent occurrence. The O9 serogroup strains were located in phylogroups A, B1, B2 and C with the exception of one O9:NM strain that was located in phylogroup B2, while the majority of serotypes were located in commensal groups. This was similar to the O104 strains given that they showed the presence of the *stx1*, *aggR* and *fimH* genes, and to a lesser extent *eae* and *sat*, which were determined as EAEC and STEC/EAEC. Miko and Delannoy [38,42] reported that O9 serotype strains presented the K9 capsular antigen but lacked the *stx* gene. However, in a previous study [40], strains with different serotypes of the *E*. *coli* O9 serogroup were located in commensal groups A and B1 and presented the *eae* and *aggR* genes. The *aggR* gene was present in strains from our study, and previous reports suggest that the strains of serotype O9 isolated from humans and animals were STEC no-O157 [48]. In the case of strains from humans, they were related to HUS [58].

Results for O104 showed antimicrobial resistance mainly to NA, TE and SXT, while O9 showed resistance to SXT and TE. This pattern of resistance was similar to that reported for *E*. *coli* O157 strains isolated from different sources, and STEC no-O157 strains isolated from dairy cattle [40,59]. However, this pattern of resistance for O104 strains was different to that reported for *E*. *coli* O104:H4 strains from the German outbreak. These strains were characterized by producing extended spectrum b-lactamase (CTX-M-15) and being resistant to ampicillin, third generation cephalosporins, nalidixic acid, SXT and TE, but sensitive to fluoroquinolones [5,12]. With respect to the O9 strains, they presented differences from isolated O9 strains from dairy cattle raised in Mexico [40]. The latter were characterized as being multi-resistant to antimicrobials, which is different from the O9 strains in this study that presented resistance against SXT and TE only.

In general, electrophoretic analysis of the O104 and O9 strains showed them to be diverse and heterogeneous, and there was no grouping by origin, serotype or phylogenetic groups. However, it is important to note that the two O104:H4 strains from the SSI presented an electrophoretic profile similar to that of strains with genes from the EAEC group, locating them in the same subgroup as an O104:H4 strain isolated from Bangladesh in 2009 and two strains isolated in 1986 and 1996 in Mexico with an O9:H4 and O9:H- serotype, respectively. The similarity between the Bangladeshi and Mexican strains and the O104:H4 strains from the SSI was 93.3% and 87.8%, respectively. This narrow location could indicate a clonal relationship between these strains, which would be interesting in order to determine if they could have a common origin.

Another interesting result was the location of the O9:H4 and O9:H- strains isolated in Mexico and Egypt, together with the ECOR27 (O104:H21) strain, which was isolated from a Giraffe in a zoo in Washington, United States [21,45]. In the referenced study, the serotype was reported as being O104:NM [45], but in our study using the serological typing system established in our laboratory, the serotype was identified as O104:H21, which corresponds with reports made by Johnson and Amor [44,45].

The ECOR28 (O104:H2) strain was isolated from a woman in Iowa, United States [21], and the serotyping results corresponded with other studies [44,45] reporting that no virulence genes were detected, similar to serotype O104:H2 isolated from human diarrhea lacking *stx* genes that was reported by Miko [41].

It is noteworthy that the ECOR26 strain with an O104:H21 serotype was isolated from a child in the United States, and a strain of O104:H4 isolated in Bangladesh in 2009 had a similarity of 93.0% despite being isolated in different geographical locations, years apart, and having distinct serotypes. However, clonally they are very close, which could indicate a wide geographical circulation of these strains.

## 5. Conclusions

Our study reports *E. coli* O104 and O9 strains isolated from different geographical locations and time periods, which present genes from various DEC groups indicating genetic plasticity and horizontal gene transfer. In addition, the results show the ability for recombination of these microorganisms in order to become incorporated into the genetic material of the genome of other DEC groups [60,61,62]. The study also reveals the importance of *E*. *coli* serotyping, which in conjunction with genotyping methods could be used in epidemiological surveillance of *E. coli* outbreaks given the wide distribution of strains with pathogenic potential.

## Figures and Tables

**Table 1:** Primers used to identify virulence genes of DEC groups and phylogenetic groups.

Genes	Nucleotide Sequence 5′-3′	Product Size (pb)	Reference
*eae* universal	F: CCC GAA TTC GGC ACA AGC ATA AGC R: CCC GGA TCC GTC TCG CCA GTA TTC	863	[26]
*Stx1*	F: GTA CGG GGA TGC AGA TAA ATC GC R: AGC AGT CAT TAC ATA AGA ACG YCC ACT	209	[27]
*Stx2*	F4: GGC ACT GTC TGA AAC TGC TCC TGT R 1: AAT AAA CTG CAC TTC AGC AAA TCC	625	
	F4-f: CGC TGT CTG AGG CAT CTC CGC T R1e/f: TAA ACT TCA CCT GGG CAA AGC C	627	
*hlyA*	F: GGT GCA GCA GAA AAA GTT GTA G R: TCT CGC CTG ATA GTG TTT GGT A	1551	[28]
*aggR*	F: CTA ATT GTA CAA TCG ATG TA R: ATG AAG TAA TTC TTG AAT	308	[29]
*aapA*	F: CTT TTC TGG CAT CTT GGG T R: GTA AC AAC CCC TTT GGA AGT	232	
*aatA*	F: ATG TTA CCA GAT ATA AAT ATA G R: CAT TTC CCC TGT ATT GGA AAT G	1064	[30]
*aaiC*	F: ATT GTC CTC AGG CAT TTC ACA CG R: ACA CCC CTG ATA AAC AA	215	[31]
*sat*	F: GGTGAGTCCGGTGCATGGGC R: CAAGTTCCGCCTGCGGCTCA	412	[32]
*fim H*	F: TGCAGAACGGATAAGCCGTGG R: GCAGTCACCTGCCCTCCGGTA	508	[33]
*arpA*	F: AAC GCT ATT CGC CAG CTT GC R-TCT CCC CAT ACC GTA CGC TA	400	[34]
*chuA*	F: ATG GTA CCG GAC GAA CCA AC R: TGC CGC CAG TAC CAA AGA CA	288	
*yjaA*	F: CAA ACG TGA AGT GTC AGG AG R: AAT GCG TTC CTC AAC CTG TG	211	
*TspE4.C2*	F: CAC TAT TCG TAA GGT CAT CC R: AGT TTA TCG CTG CGG GTC GC	152	

**Table 2:** Phylogenetic groups and virulence factors in *E*. *coli* O104 strains.

Location	Year of Isolation	Origin	*E*. *coli* Serotypes	* Phylogenetic Group	Strains	Virulence Genes										
						*eae*	*stx1*	*stx2*	*hlyA*	*aggR*	*app*	*aatA*	*aaiC*	*sat*	*fimH*	Pathotype
Mexico	1998	** IMSS	O104:H4	B1	1	1	−	−	−	1	1	1	−	−	1	a-EPEC/EAEC
	2003		O104:H7		1	1	1	−	−	−	−	−	−	−	1	STEC
			O104:H12		1	−	−	1	−	−	−	−	−	−	1	STEC
			O104:H12	A	2	2	2	−	−	−	−	−	−	−	2	STEC
Egypt	1996	Egypt	O104:H4	A	1	1	1	−	1	1	1	1	1	1	−	STEC/EAEC
Argentina	2003	Argentina	O104:H12	A	3	3	2	3	2	−	−	−	−	−	3	STEC
			O104:H4		1	1	1	−	1	−	1	−	1	1	−	STEC
Bangladesh	2009	*** ICDDR,B	O104:H4	A	1	1	1	−	−	1	1	1	1	1	−	STEC/EAEC
			O104:H12		1	1	1	−	−	1	1	1	1	1	−	STEC/EAEC
			O104:H4	B1	1	1	1	−	1	1	1	1	1	1	−	STEC/EAEC
N (%)					13	12 (92)	10 (77)	4 (31)	5(39)	5 (39)	6 (46)	5 (39)	5 (39)	5 (39)	8 (62)	

* Group A: *arpA*+, *chuA*-, *yjaA*-, *TspE4.C2*-; group B1, *arpA*+, *chuA*-, *yjaA*-, *TspE4.C2C*+; group B2: *arpA-*, *chuA+*, *yjaA+*, *TspE4.C2C+*; group C: *arpA*+, *chuA*-, *yjaA*+, *TspE4.C2C*-. ** IMSS: Instituto Mexicano del Seguro Social (Mexican Institute of Social Security). *** International Centre for Diarrhoeal Disease Research, Bangladesh.

**Table 3:** Diversity of diarrheagenic groups in *E*. *coli* O9 strains.

Location	Isolation Year	Origin	Serotype	Phylogenetic Group *	Strains	Virulence Genes								Pathotype
						*eae*	*stx1*	*hlyA*	*aggR*	*app*	*aatA*	*sat*	*fimH*	
Mexico	1986, 1987	Tlaltizapan ^1^, Mor.	O9:NM	A	5	−	5	1	−	3	−	−	4	STEC
	1986		O9:NM		1	−	−	−	1	1	−	−	1	EAEC
			O9:H9		2	−	2	−	2	−	2	−	2	STEC/EAEC
			O9:H10		1	−	1	−	1	−	−	−	1	STEC/EAEC
	1987		O9:H11	B1	2	−	2	−	−	-	−	2	2	STEC
	1986		O9:NM	C	2	−	2	2	−	2	−	−	2	STEC
	1996	** IMSS	O9:H4	A	1	−	1	−	−	-	−	−	1	STEC
			O9:H33		2	2	2	−	−	-	−	2	2	STEC
			O9:H4		3	−	3	−	3	2	1	−	3	STEC/EAEC
			O9:H33		2	2	−	−	−	-	−	2	2	a-EPEC
			O9:NM		1	−	1	−	1	-	−	−	1	STEC/EAEC
			O9:H21	B1	1	−	−	1	−	-	−	−	1	ND
	2000		O9:H25		3	−	3	−	3	−	−	−	3	STEC/EAEC
			O9:H25		4	−	−	2	4	−	−	−	4	EAEC
Egypt	1997	Egypt	O9:NM	B2	1	−	−	−	1	-	−	−	1	EAEC
			O9:NM	A	1		1		1				1	STEC/EAEC
	1999		O9:NM	A	1	−	−	−	1	-	−	−	1	EAEC
			O9:H9		1	−	−	−	1	-	−	−	1	EAEC
N (%)					34	4 (12)	23 (68)	6 (18)	19 (56)	8 (24)	3 (9)	6 (18)	33 (97)	

^1^ Cravioto A, [20]. * Group A: *arpA*+, *chuA*-, *yjaA*-, *TspE4.C2*-; Group B1, *arpA*+, *chuA*-, *yjaA*-, *TspE4.C2C*+; group B2: *arpA-*, *chuA+*, *yjaA+*, *TspE4.C2C+*; group C: *arpA*+, *chuA*-, *yjaA*+, *TspE4.C2C*-. All O9 strains were negative for *stx2* and *aaiC.* ** IMSS: Instituto Mexicano del Seguro Social (Mexican Institute of Social Security).

**Table 4:** Serotypes, phylogenetic groups and virulence genes in *E*. *coli* O104 ECOR and references strains.

Origin	Year of Isolation	*E*. *coli* Serotypes	Phylogenetic Group	Strains	Virulence Genes									
					*eae*	*stx1*	*hlyA*	*aggR*	*app*	*aatA*	*aaiC*	*sat*	*fimH*	Pathotype
ECOR26	1984	O104:H21	A	1	1	−	1	−	−	−	−	−	1	a-EPEC
ECOR27		O104:H21		1	−	1	1	−	−	−	−	−	1	STEC
ECOR28		O104:H2		1	−	−	1	−	−	−	−	−	1	ND
SSI	2013	O104:H4	D	1	−	−	−	1	1	1	1	1	−	EAEC
	2018	O104:H4	B1	1	−	−	−	1	1	1	1	1	−	EAEC
GBRU	1999	O9:H12	A	1	−	−	−	1	-	−	−	−	1	EAEC
ECOR16	1984	O9:H10		1		−	−	−	−	−	−	−	1	ND
N (%)				7	1 (14)	1 (14)	3 (43)	3 (43)	2 (29)	2 (29)	2 (29)	2 (29)	5 (71)	

All the reference strains did not present the *stx2*. ECOR: *Escherichia coli* reference. SSI: Statens Serum Institute. GBRU: Gastrointestinal Bacteria Reference Unit, Public Health England, London, UK. ND: Not determined.

**Table 5:** Resistance patters of *E*. *coli* O104 strains.

Country	Year of Isolation	Serotype	Strains	Number of Strains					Antimicrobial Resistance
				ATM	CAZ	NA	TE	SXT	
Mexico	1998	O104:H4	1				R		1
	2003	O104:H12	2			R	R		2
		O104:H12	1	R	R	R			3
Egypt	1996	O104:H4	1					R	1
Bangladesh	2009	O104:H4	1			R			1
		O104:H4	2			R	R		2
SSI	2013–2018	O104:H4	2				R	R	2
N (%)			10	1 (10)	1 (10)	7 (70)	7 (70)	3 (30)	

ATM: Aztreonam. CAZ: Ceftazidime. SXT: Trimethoprim/sulfamethoxazole. NA: Nalidixic Acid. TE: Tetracycline.

**Table 6:** Resistance patters of *E*. *coli* O9 strains.

Country	Year of Isolation	Origin	Serotype	Strains	Antimicrobial Resistance				Total Antimicrobials
					ATM	NA	TE	SXT	
Mexico	1986–1987	Tlaltizapan Mor., ^1^	O9:NM	2				R	1
			O9:NM	1			R		1
			O9:NM	2			R		2
			O9:H10	1			R		1
			O9:H11	2			R		2
	1996	** IMSS	O9:NM	1				R	1
			O9:H33	2		R	R		2
			O9:H4	1			R		1
			O9:H33	1			R	R	2
	1999		O9:H-	1	R				1
	2000		O9:H25	2			R		1
			O9:H25	1				R	1
			O9:H25	2			R	R	2
Egypt	1999		O9:NM	1			R		1
			O9:NM	1			R	R	2
Total N (%)				21 (62)	1 (3)	1 (3)	11 (32)	6 (18)	

^1^ Cravioto A, [20]. ** IMSS: Instituto Mexicano del Seguro Social (Mexican Institute of Social Security). ATM: Aztreonam. NA: Nalidixic Acid. TE: Tetracycline. SXT: Trimethoprim/sulfamethoxazole.

## Data Availability

Not applicable.

## Supplementary Materials

The following are available online at https://www.mdpi.com/article/10.3390/microorganisms9112227/s1, Table S1: Agglutination titers of anti-O9 and O104 sera without unabsorbed and absorbed.
