# Supplementary material for: Diversity of Potentially Pathogenic Escherichia coli O104 and O9 Serogroups Isolated before 2011 from Fecal Samples from Children from Different Geographic Regions
Source: Microorganisms. 2021 Oct 26;9(11):2227. doi: 10.3390/microorganisms9112227 (PMC8619403; doi:10.3390/microorganisms9112227)
Supplement: Supplementary file 1 [file microorganisms-09-02227-s001.zip › microorganisms-1406310-supplementary.pdf]

Supplementary Table S1. Agglutination titers of anti-O9 and O104 sera without unabsorbed and absorbed.

Antigens	Titers of unabsorbed sera		Titers of absorbed sera	
	Anti-O9	Anti-O104	Anti-O9	Anti-O104
Absorbed with			*E. coli* O104	*E. coli* O9
E. coli O9	1:1600	1:200	1:400	-
E. coli O104	1:400	1:1600	-	1:400

Serum was absorbed with boiled antigen for 1 h.
